# Polyphenols journey through blood-brain barrier towards neuronal protection

**DOI:** 10.1038/s41598-017-11512-6

**Published:** 2017-09-13

**Authors:** I. Figueira, G. Garcia, R. C. Pimpão, A. P. Terrasso, I. Costa, A. F. Almeida, L. Tavares, T. F. Pais, P. Pinto, M. R. Ventura, A. Filipe, G. J. McDougall, D. Stewart, K. S. Kim, I. Palmela, D. Brites, M. A. Brito, C. Brito, C. N. Santos

**Affiliations:** 1 Instituto de Tecnologia Química e Biológica – António Xavier, Universidade Nova de Lisboa, Av. da República, EAN, 2781-901 Oeiras, Portugal; 2 Instituto de Biologia Experimental e Tecnológica, Apartado 12, 2781-901 Oeiras, Portugal; 3 Instituto Gulbenkian de Ciência, Rua da Quinta Grande, 6, 2780-156 Oeiras, Portugal; 4 Escola Superior Agrária, Instituto Politécnico de Santarém, Qta do Galinheiro, Santarém, Portugal; 5 Medical Department, Grupo Tecnimede, 2710-089 Sintra, Portugal; 6 The James Hutton Institute, Invergowrie, Dundee, DD2 5DA Scotland United Kingdom; 7 Engineering and Physical Sciences, Heriot Watt University, Edinburgh, EH14 4AS Scotland United Kingdom; 8 NIBIO, Norwegian Institute of Bioeconomy Research, Pb 115, NO-1431 Ås, Norway; 9 Division of Infectious Diseases, Johns Hopkins University School of Medicine, 600 North Wolfe Street Park 256, Baltimore, MD21287 USA; 10 Research Institute for Medicines (iMed.ULisboa), Faculty of Pharmacy, Universidade de Lisboa, Av. Prof. Gama Pinto, 1649-003 Lisbon, Portugal; 11 Department of Biochemistry and Human Biology, Faculty of Pharmacy, Universidade de Lisboa, Av. Prof. Gama Pinto, 1649-003 Lisbon, Portugal

**Keywords:** Mechanism of action, Small molecules

## Abstract

Age-related complications such as neurodegenerative disorders are increasing and remain cureless. The possibility of altering the progression or the development of these multifactorial diseases through diet is an emerging and attractive approach with increasing experimental support. We examined the potential of known bioavailable phenolic sulfates, arising from colonic metabolism of berries, to influence hallmarks of neurodegenerative processes. *In silico* predictions and *in vitro* transport studies across blood-brain barrier (BBB) endothelial cells, at circulating concentrations, provided evidence for differential transport, likely related to chemical structure. Moreover, endothelial metabolism of these phenolic sulfates produced a plethora of novel chemical entities with further potential bioactivies. Pre-conditioning with phenolic sulfates improved cellular responses to oxidative, excitotoxicity and inflammatory injuries and this attenuation of neuroinflammation was achieved *via* modulation of NF-κB pathway. Our results support the hypothesis that these small molecules, derived from dietary (poly)phenols may cross the BBB, reach brain cells, modulate microglia-mediated inflammation and exert neuroprotective effects, with potential for alleviation of neurodegenerative diseases.

## Introduction

With increased life expectancy^1^, the world’s population is getting older with concomitant incidence of age-related diseases, like Alzheimer’s and Parkinson’s disease, the two most common age-related neurodegenerative disorders^2^. Current therapies only alleviate physical complications, being unable to abolish the pathology, comprising a huge burden to the society^3^. The difficulty in finding drugs to treat neurodegenerative disorders can be explained by the multitude of factors that lead to disease phenotype and effective treatments will need to be muli-faceted^4^.

Through the past decades, several epidemiological studies have revealed that (poly)phenol-rich diets, including *e.g.* fruits and vegetables, can provide beneficial effects in humans^5, 6^, preventing degenerative disorders and cognitive decline^7, 8^. (Poly)phenols are described as pleiotropic and may act against several disease-relevant biological pathways^9, 10^. Nutritional studies have also demonstrated significant cognitive benefits and neuroprotective potential of (poly)phenols^11–14^. Berries are amongst the most promising fruits as sources of (poly)phenols with these health benefits^11, 15–18^.

Despite the accumulating evidence of beneficial effects, the basic mechanism of action of (poly)phenols remain to be elucidated^19^. Both indirect actions through peripheral effects (*e.g*. enhancement of cerebrovascular blood flow) and direct actions inside the brain (*e.g*. through activation of receptors, neurotrophins and modulation of signaling pathways) have been suggested as potential mechanisms. Most *in vitro* mechanistic studies with (poly)phenols have used pure components and do not consider their metabolism and bioavailability. Therefore, the effects reported do not necessarily relate to what may occur *in vivo* as (poly)phenol metabolites present in circulation result from extensive conjugation due to digestion, hepatic and colonic metabolism, and usually differ from their native dietary compounds^20^. In addition, the concentration ranges used are much higher than the levels of circulating “bioavailable” metabolites.

Recent studies demonstrate that, after intestinal absorption, some (poly)phenol metabolites can reach concentrations in the bloodstream that can exert effects *in vivo* ^21, 22^. Nevertheless, the effective brain uptake of these (poly)phenols metabolites, with possible direct neuroprotective potential, is still regarded with some reservations and the true mechanisms by which they may permeate the blood-brain barrier (BBB) are not fully understood.

The BBB is a dynamic interface that limits and regulates molecular exchanges between the blood and the neuronal tissue or its fluid spaces, having a crucial role in providing nutrients and non-nutrients (such as (poly)phenols), and controlling the access of compounds to the brain^23, 24^. Assays with mammals revealed that (poly)phenols and their metabolites can enter the brain at measurable levels, supporting their direct action in a neurological context^25–27^. Youdim and co-workers have also begun to elucidate the mechanisms of permeation of (poly)phenols through the BBB^28, 29^. Nevertheless, it is not yet completely clear whether the primary route by which (poly)phenol metabolites cross the BBB is by simple diffusion or by specific carrier-mediated transport. Moreover, there is also limited knowledge of how (poly)phenol structure influences their brain bioavailability. Additionally, little is known about their further metabolism in the brain.

In previous work we identified new bioavailable (poly)phenol metabolites in urine and human plasma after consumption of a mixed berry puree^22, 30^ and determined their circulating concentrations. These bioavailable metabolites circulate in micromolar concentrations whereas their parent compounds are undetected^22^. Here we report, for the first time, that these metabolites are able to cross the BBB endothelium at physiologically relevant concentrations. Moreover, endothelial cells metabolize these metabolites into novel components, which provides a new array of candidate brain-available metabolites never previously studied. We also demonstrate that these (poly)phenol metabolites exert beneficial effects in different neuronal systems (*e.g*. cell lines, primary cultures and a three-dimensional human cell model), with different degrees of complexity and in response to different damages. The (poly)phenol metabolites attenuated neuro-inflammatory processes *via* regulation of nuclear factor (NF)-κB translocation into the nucleus and modulation of IκBα levels.

## Results

### Bioavailable (poly)phenol metabolites are transported across the BBB endothelium

The transport of bioavailable (poly)phenol metabolites across the BBB was evaluated in an immortalized human brain microvascular endothelial cell (HBMEC) line that mimics endothelial cells of brain capillaries, considered the anatomical basis of the BBB^23, 31^.

(Poly)phenol metabolites known to be bioavailable were synthetized and tested at 5 μM as they have been quantified at physiological levels ranging from 0.3–12 µM in plasma^22^ (Table 1). At this range of concentrations, it was verified that there are no adverse effects on cellular viability in HBMEC line up to 24 h of incubation for all compounds (Supplementary Fig. S1).

**Table 1:** Human bioavailable (poly)phenol metabolites. (Poly)phenol metabolites nomenclature, abbreviation, chemical structure and C_max_ are presented.

Nomenclature	Abbreviation	Structure	C_max_ (µM)^1^
Catechol-*O*-sulfate	Cat-sulf		12.2 ± 5.9
Pyrogallol-*O-*sulfate^2^	Pyr-sulf		11.4 ± 6.7 and 0.65 ± 0.3
1-*O*-methylpyrogallol-*O*-sulfate^3^	1-MePyr-sulf		2.88 ± 1.8
4-*O*-methylgallic acid-3-*O*-sulfate	4-MeGA-sulf		2.03 ± 1.1
2-*O*-methylpyrogallol-1-*O*-sulfate	2-MePyr-sulf		1.97 ± 1.0
Vanillic acid 4-*O*-sulfate	VA-sulf		1.34 ± 1.3
4-Methylcatechol *O*-sulfate^4^	4-MeCat-sulf		0.64 ± 0.5
4-*O*-methylgallic acid	4-MeGA		0.30 ± 0.1

^1^C_max_ values were determined by evaluation of human plasma samples in a previous work^22^.

^2^Mixture of two compounds in approximately similar proportion, 53% of Pyrogallol-2-*O-*sulfate (Pyr-2-sulf) and 47% of Pyrogallol-1-*O-*sulfate (Pyr-1-sulf), and for each of them C_max_ was obtained since they are chromatographically distinguishable.

^3^Mixture of two compounds in approximately similar proportion, 56% of 1-*O*-methyl pyrogallol-2-*O*-sulfate (1-MePyr-2-sulf) and 44% of 1-*O*-methyl pyrogallol-3-*O*-sulfate (1-MePyr-3-sulf), chromatographically indistinguishable.

^4^Mixture of two compounds (4-MeCat-1-sulf and 4-MeCat-2-sulf) present at 64% and at 36%, respectively, and chromatographically indistinguishable.

**Figure 1:**
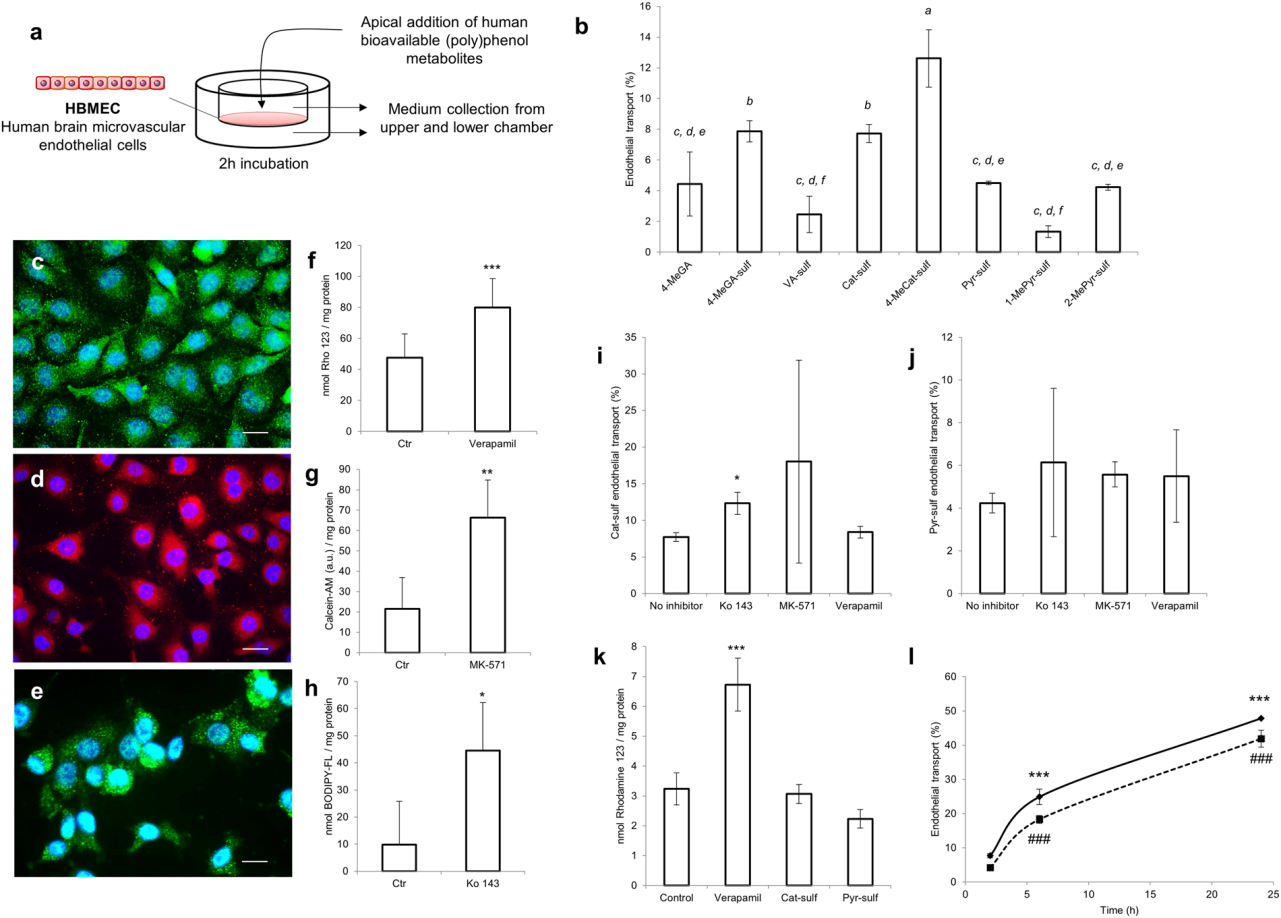
Blood-brain barrier transport of human bioavailable (poly)phenol metabolites. (**a**) Schematic experimental design used to assess (poly)phenol metabolites transport across the BBB. (**b**) Endothelial transport of human bioavailable polyphenol metabolites after 2 h of incubation. Endothelial transport was evaluated by LC-Orbitrap MS and is presented as percentage (%) determined by the ratio of the lower compartment concentration and the sum of the upper and lower compartments concentrations. Statistical differences for p < 0.01 are denoted from *a-f*. (**c–e**) Immunofluorescence detection of major efflux transporters in HBMEC line: (**c**) P-gp, in green, (**d**) MRP1, in red and (**e**) BCRP, in green. Nuclei stained with DAPI (blue). Scale bar: 40 µm. (**f–h**) HBMEC intracellular accumulation of specific efflux transporters’ substrates in the presence of the respective inhibitors: (**f**) 1 µM of verapamil (P-gp inhibitor), (**g**) 1 µM MK-571 (MRP1 inhibitor) or (**h**) 1 µM of Ko 143 (BCRP inhibitor). Statistical differences are denoted as ***p < 0.001, **p < 0.01 and *p < 0.05 relatively to control cells. Endothelial transport of (**i**) Cat-sulf and (**j**) Pyr-sulf when co-incubated with efflux transporters inhibitors. Statistical differences in the presence of inhibitors are denoted as *p < 0.05 relatively to “No inhibitor”. (**k**) P-gp substrate accumulation for Cat-sulf and Pyr-sulf compared with verapamil. Intracellular accumulation of P-gp substrate, Rhodamine 123 was evaluated after pre-incubation of cells with the bioavailable (poly)phenol metabolites. Statistical differences are denoted as ***p < 0.001 relatively to control. (**l**) Endothelial transport of Cat-sulf (solid line) and Pyr-sulf (dashed line) along time. Statistical differences along time are denoted as ***p < 0.001, relatively to 2 h of incubation in Cat-sulf, or ^###^p < 0.001, relatively to 2 h of incubation in Pyr-sulf. All values are means ± SD, n=3.

We used the well-validated confluent HBMEC two chamber BBB model^32^ to investigate endothelium transport with metabolite quantification by Orbitrap LC-MS techniques. (Poly)phenol metabolites were added in the upper chamber and their putative transport through the BBB endothelium assessed after 2 h of incubation (Fig. 1a).

Different concentrations of each compound were detected in the upper and lower chambers, suggesting a differential transport of the metabolites (Fig. 1b). For gallic acid (GA) derivatives, the combination of both methylation and sulfation, 4-*O*-methylgallic acid-3-*O*-sulfate (4-MeGA-sulf), increased BBB permeation compared with 4-methylgallic acid (4-MeGA). Intriguingly, 4-methylcatechol *O*-sulfate (4-MeCat-sulf) was more effectively transported than catechol-*O*-sulfate (Cat-sulf) (Fig. 1b). Moreover, in the case of pyrogallol-*O*-sulfate (Pyr-sulf), the position of the methylation influenced BBB passage; 1-*O*-methylpyrogallol-*O*-sulfate (1-MePyr-sulf) was less effectively transported whereas 2-*O*-methylpyrogallol-1-O-sulfate (2-MePyr-sulf) isomer was transported as effectively as Pyr-sulf. Additionally, Pyr-sulf consists in a mixture of isomers (Pyr-1-sulf and Pyr-2-sulf) (Table 1, see note 2) and we detected only Pyr-2-sulf in the basolateral compartment of cells.

No statistically significant difference was observed in the transport percentage when 5 or 10 µM of the metabolites was applied (Supplementary Fig. S2).

### *In silico* modeling of metabolite properties

Accessibility to the brain may be dependent to some extent on the structural properties of metabolites. An *in silico* prediction of BBB permeability of the various metabolites was carried out using the QikProp software. QikProp predicts the ability of specific molecules to cross the BBB (Table 2). Estimated QikProp descriptors were within the range of values for 95% of known drugs (according to Schrödinger software, https://www.schrodinger.com/qikprop) and did not vary much between the different compounds, which is reasonable considering their structural similarity.

**Table 2:** *In silico* calculations of BBB permeation for human bioavailable (poly)phenol metabolites.

Molecule	Dipole	Volume (cm^3^)	donorHB	accptHB	QPlogPo/w	QPPCaco (nm/s)	QPlogBB	QPPMDCK (nm/s)	PSA (Å^2^)	QPlogKhsa
Cat-Sulf	8.5	541.2	2	5	−0.06	33.6	−1.24	16.6	89.4	−1.00
Pyr-2-sulf	10.5	549.2	3	6	−0.37	28.0	−1.38	13.6	96.7	−1.05
Pyr-1-sulf	9.4	586.8	3	6	−0.48	12.1	−1.70	5.5	110.9	−1.00
1-MePyr-2-sulf	5.9	627.0	2	6	0.24	48.4	−1.21	24.3	98.8	−0.97
1-MePyr-3-sulf	5.8	625.8	2	6	0.20	45.1	−1.23	22.5	100.1	−0.97
4-MeGA-sulf	4.8	676.8	2	8	−0.29	1.9	−1.90	0.9	128.4	−1.24
2-MePyr-sulf	2.9	635.1	2	6	0.25	43.3	−1.26	21.5	100.3	−0.96
VA-sulf	4.7	665.7	2	7	0.11	3.5	−1.73	1.8	114.1	−1.22
4-MeCat-1-sulf	9.0	625.8	2	5	0.34	33.5	−1.31	16.5	89.5	−0.85
4-MeCat-2-sulf	8.7	623.2	2	5	0.35	35.8	−1.27	17.7	89.5	−0.86
4-MeGA	2.9	573.4	3	4	0.23	34.7	−1.20	16.7	100.8	−0.85

QikProp descriptors were obtained for each metabolite, namely dipole, volume, donor HB, accptHB, QPlogPo/w, QPPCaco, QPlogBB, QPPMDCK, PSA, QPlogKhsa.

DonorHB – number of donor hydrogen bonds; accptHB – number of acceptor hydrogen bonds; QPlogPo/w – predicted octanol/water partition coefficient (for 95% of known drugs values range between −2.0 and 6.5); QPPCaco – predicted apparent Caco-2 cell permeability (non-active gut-blood barrier transport; <25 poor,>500 great); QPlogBB – predicted brain/blood partition coefficient (for 95% of known drugs values range between −3.0 and 1.2); QPPMDCK – predicted apparent MDCK (Madin-Darby Canine Kidney Epithelial Cells- consider a good model to mimic the BBB) cell permeability (non-active blood-brain barrier transport; <25 poor, >500 great); PSA – Van der Waals surface area of polar nitrogen and oxygen atoms and carbonyl carbon atoms (for 95% of known drugs values range between 7.0 and 200.0); QPlogKhsa – prediction of binding to human serum albumin (for 95% of known drugs values range between −1.5 and 1.5).

None of the compounds tested were predicted to have CNS activity only assuming passive BBB diffusion (CNS activity: −2).

For the octanol/water partition coefficient predictor (QPlogPo/w), the (poly)phenol metabolites were at the lower range of the recommended values, that indicates poor passive diffusion. Interestingly, the metabolites with a methyl group on (4-MeCat-1-sulf, 4-MeCat-2-sulf, 2-MePyr-sulf, 1-MePyr-2-sulf, 1-MePyr-3-sulf) gave the highest values (Table 2). Moreover, 4-MeCat-sulf and Cat-sulf gave the lowest PSA values (Table 2), which were below the recommended threshold of 90 Å^2^, which also favors possible﻿ passive BBB permeation^33^.

Another important descriptor that predicts the brain/blood partition is the QPlogBB. The calculated values for our molecules, although within the recommended values for brain drugs, were not high. Additionally, the predicted apparent MDCK cell permeability was very low for all compounds. The more charged compounds, such as 4-MeGA-sulf and VA-sulf have the lowest values (Table 2).

An important feature that could influence compounds ability to be actively transported through the BBB is their ability to establish hydrogen bonds with other functional groups. The carboxylic acids (VA-sulf, and 4-MeGA-sulf) have relatively high cumulative values as hydrogen exchangers (*e.g*. donorHB + accptHB). Moreover, the other carboxylic acid, 4-MeGA had highest value of QPlogKhsa, *i.e.* it is more likely to bind to human serum albumin.

Overall, QikProp analysis suggested that none of the metabolites would be able to cross the BBB endothelium by passive permeation but some form of active transport could be involved.

### HBMEC contains functionally active efflux transporters

Other factors could limit the levels of metabolites inside the brain besides transport mechanisms, such as efflux systems. To our knowledge, HBMEC cells have not yet been characterized for the expression or activity of major efflux transporters. By immunofluorescence, we detected the presence of the three major membrane ATP-binding cassette protein (ABC)-type efflux transporters in HBMEC cells (Fig. 1c–e), previously described to be present in brain endothelial cells and known for their broad substrate specificity; P-glycoprotein (P-gp, *ABCB1*), multidrug resistance-associated protein 1 (MRP1, *ABCC1*) and breast cancer resistance protein (BCRP, *ABCG2*)^34^. Functional activities of P-gp, MRP1 and BCRP were validated using substrate accumulation assays (Fig. 1f–h) and specific inhibitors for each transporter. Verapamil was used as a P-gp inhibitor and 1 µM was sufficient to reduce its detoxifying capacity assessed by the intracellular accumulation of its substrate, Rhodamine 123 (Fig. 1f). We also confirmed the functional detoxifying activity of MRP1 (Fig. 1g) and BCRP (Fig. 1h), by using Calcein-AM and BODIPY-FL, respectively, as specific substrates, and MK-571 and Ko143 as the respective inhibitors.

### Bioavailable (poly)phenol metabolites are not exported to the upper side and their transport increases with time

We also assessed how these efflux transporters could influence the permeation of (poly)phenol metabolites, in particular them﻿ore ﻿abund﻿ant plasma-bioavailable metabolites Cat-sulf and Pyr-sulf^22^, into the lower compartment of the BBB *in vitro* model (Fig. 1a). Inhibition of the efflux transporters did not influence the BBB transport of Cat-sulf (Fig. 1i) and Pyr-sulf (Fig. 1j). However, BCRP may be partly involved in the efflux of Cat-sulf as there was a significant increase (p < 0.05) in its endothelial transport after treatment with BCRP specific inhibitor, Ko 143 (Fig. 1i).

To determine if the metabolites could affect cellular efflux capacity, we evaluated the functional activity of the most well-studied efflux transporter in humans, P-gp^35^. Under the same conditions that Verapamil doubled Rhodamine 123 accumulation, no statistically- significant alterations in P-gp activity were noted in the presence of Cat-sulf and Pyr-sulf at 5 μM (Fig. 1k). The same trend was observed for the other bioavailable (poly)phenol metabolites (Supplementary Fig. S3).

Transport of Cat-sulf and Pyr-sulf in the *in vitro* BBB model increased over 2, 6 and 24 h. Although, there was a significant increase in transport percentage of both Cat-sulf and Pyr-sulf with time (Fig. 1l), even after 24 h incubation, no transport of the less physiologically-representative isomer of Pyr-sulf (Pyr-1-sulf) was detected.

### HBMEC metabolize bioavailable (poly)phenol metabolites

One phenomenon that may influence endothelium transport percentage for (poly)phenol metabolites across BBB is their metabolism within the cells. To identify possible cellular metabolites, the LC-MS data for the upper and lower compartments was searched against an in-house database of predicted metabolites^22^ for compounds of predicted theoretical masses and fragmentation patterns and new putative cellular metabolites were detected. These included those arising from cellular conjugation with glucuronic acid or glutathione (Supplementary Table S1; results for the remaining metabolites are presented in Supplementary Tables S2–4).

**Figure 2:**
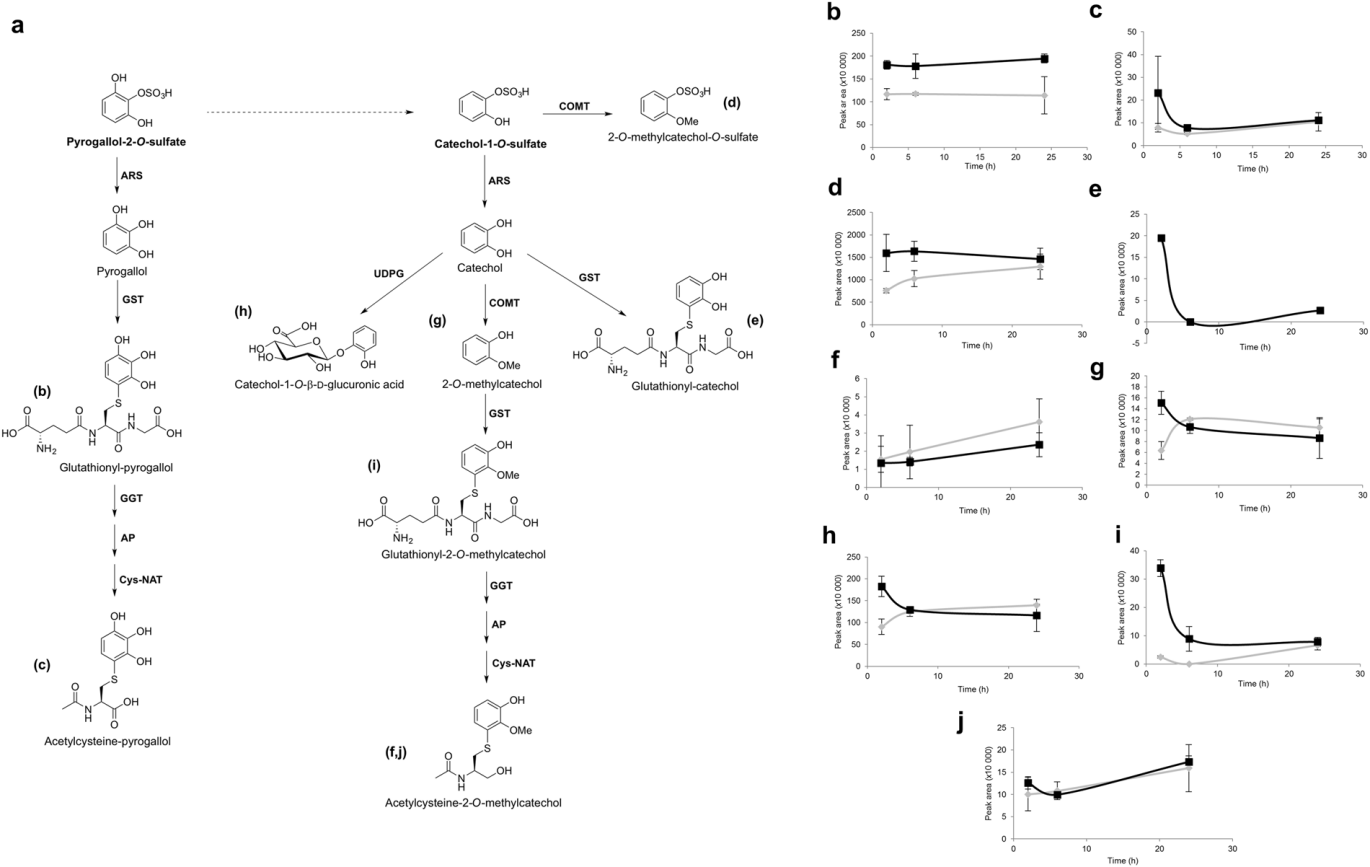
Blood-brain barrier endothelial cells metabolization of Pyr-sulf and Cat-sulf. (**a**) Putative pathways and enzymes that could be involved in endothelial metabolism into novel phenolic compounds. Proposed metabolization route of the compounds was designed based on canonical enzymatic reactions described in KEGG (Kyoto Encyclopedia of Genes and Genomes). ARS - Arylsulfatase; GST - Glutathione S-transferase; GGT - Gamma glutamyl transferase; AP - Aminopeptidase; Cys-NAT – Cysteine N-acetyl transferase; COMT - Catechol *O*-methyl transferase; UDPG – UDP-Glucuronosyl transferase. Relative quantification (peak areas) of the novel phenolic metabolites appearing in upper (grey) and lower (black) compartments along time after addition of (**b–f**) Pyr-sulf or (**g–j**) Cat-sulf in upper compartment, namely (**b**) Glutathionyl-pyrogallol, (**c**) Acetylcysteine-pyrogallol, (**d**) 2-*O*-methylcatechol-*O*-sulfate, (**e**) Glutathionyl-catechol and (**f**) Acetylcysteine-2-O-methylcatechol, (**g**) 2-*O*-methylcatechol, (**h**) Catechol-1-*O*-β-D-glucoronic acid, (**i**) Glutathionyl-2-*O*-methylcatechol, and (**j**) Acetylcysteine-2-O-methylcatechol. Note: the compound Acetylcysteine-2-O-methylcatechol was detected in both samples (panels’ f and j).

The proposed cellular pathways of Cat-sulf and Pyr-sulf metabolism were designed based on canonical enzymatic reactions described in KEGG (Kyoto Encyclopedia of Genes and Genomes, Fig. 2a). Moreover, the relative abundance of the novel metabolites (Fig. 2b–j) detected in upper or lower compartments, was assessed, which indicated that each metabolite had a different pattern with time. Interestingly, we observed that the cellular metabolites more proximate to the original metabolites (*e.g.* glutathionyl-pyrogallol, Fig. 2b) were detected in higher amounts than the ones more distant (*e.g.* acetylcysteine-pyrogallol, Fig. 2c).

### Bioavailable (poly)phenol metabolites protect brain endothelial cells and neuronal cells

Three different cell systems were selected to assess the breadth of neuroprotective potential of metabolites on neuronal excitotoxicity and oxidative stress, common hallmarks in neurodegenerative disorders^36^; a cell line representative/model of the BBB, primary cultures of mouse cerebellar granule cells, and human 3D aggregates of both neurons and astrocytes. The preventive effects of Cat-sulf and Pyr-sulf were assessed after pre-incubation at physiologically-relevant concentrations and prior to administering the specific insult.

**Figure 3:**
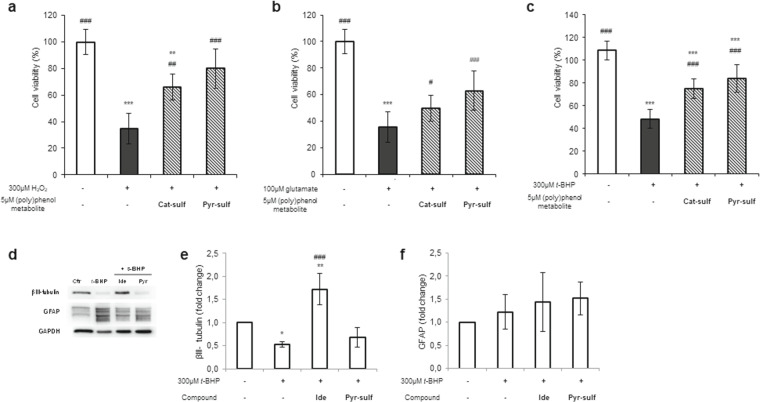
Cytoprotective potential of Cat-sulf and Pyr-sulf. (**a**) HBMEC line submitted to oxidative stress (300 µM H_2_O_2_); (**b)** primary mouse cerebellar granule cells exposed to glutamate excitotoxicity (100 µM glutamate); (**c**) 3D aggregates containing neurons and astrocytes exposed to oxidative injury (300 µM *t*-BHP). Cells were pre-incubated with 5 µM of each bioavailable polyphenol metabolite for 24 h and then injured with the respective lesion. Cell viability was assessed and is presented as percentage relatively to control. Statistical differences are denoted as ***p < 0.001, **p < 0.01 and *p < 0.05 relatively to control and as ^###^p < 0.001, ^##^p < 0.01 and ^#^ ﻿p﻿< 0.05 relatively to each lesion (H_2_O_2_, glutamate or *t*-BHP). (**d-f**) Alterations in protein markers of the neuronal (β-III tubulin) and astrocytic (GFAP) population of 3D aggregates t﻿﻿owards the *t-*BHP lesion without and with﻿ pre-incubation with idebenone (Ide), a control drug, and with P﻿yr-sulf. (**d**) Representative western blot and (**e**) β-III tubulin and (**f**) GFAP fold changes in protein levels normalized to GAPDH. Statistical differences are denoted as ***p < 0.001, **p < 0.01 and *p < 0.05 relatively to control and as ^###^p < 0.001 relatively to *t*-BHP. Western blots were analyzed under the same experimental conditions. Data are presented as the means ± SD, n = 3.

As one of the first lines of defense, endothelial cells of the BBB prevent damage to the brain from constant insults^37^. The HBMEC line treated with 300 µM of hydrogen peroxide for 24 h was used as a model of brain endothelial injury. Pre-incubation of Cat-sulf and Pyr-sulf prior to injury improved cell viability, with Pyr-sulf being more effective than Cat-sulf (Fig. 3a), maintaining cell viability at untreated cells levels. The other less abundant human bioavailable metabolites also caused significant cytoprotective effects to different extents (Supplementary Fig. S4).

Cat-sulf and Pyr-sulf were also assessed in a classic model of excitotoxicity^38^, in which primary cultures of mouse cerebellar granule cells were challenged with glutamate. Both Cat-sulf and Pyr-sulf improved cell viability of cerebellar granular cells under glutamate-induced excitotoxicity (Fig. 3b), again with Pyr-sulf being more effective.

Pre-incubation of Cat-sulf and Pyr-sulf also caused significant neuroprotection in 3D aggregates against *tert*-butyl hydroperoxide (*t*-BHP)-induced injury (Fig. 3c). Both sulfates were equally protective and gave the same level of protection as conferred by idebenone^39^, a drug used for of Alzheimer’s disease known to boost mitochondrial ATP production^40^. Indeed, the other bioavailable metabolites also caused significant neuroprotective effects under the same conditions (Supplementary Fig. S4).

In all 3 cell systems, Pyr-sulf appeared to be more effective than Cat-sulf; therefore we decided to explore its mechanisms in greater depth using the 3D aggregates, a more physiologically-relevant system, and we compared it against the positive control idebenone^41^. Levels of β-III tubulin and glial fibrillary acidic protein (GFAP) were studied as markers of neurons and astrocytes, respectively (Fig. 3d–f). The 3D aggregates challenged with *t*-BHP showed a significant decrease in β-III tubulin protein levels but not GFAP protein levels, an effect counteracted by idebenone, which increased β-III tubulin protein levels. Pyr-sulf pre-incubation was ineffective in altering *t*-BHP induced changes in β-III tubulin levels (Fig. 3d–f). Therefore, this suggests that *t*-BHP treatment has a greater impact on neuronal cells and this effect could not prevented by Pyr-sulf pre-treatment. Indeed, this cell-specific effect was hidden in the overall neuroprotective effect on total cell viability noted in Fig. 3c.

After 24 h of pre-incubation with Pyr-sulf before the *t*-BHP insult, expression of *SOD1*, *GPX1* and *GSR*, important antioxidant enzymes, were not significantly different from control, suggesting that the cells may already have returned to basal levels (Supplementary Fig. S5). Interestingly, *t*-BHP treatment alone significantly increased *SOD1* expression. We also did not see effects on the complex subunits from the mitochondrial respiratory chain that could be associated with Pyr-sulf protection (Supplementary Fig. S5). When we looked for key determinants in apoptosis. Pyr-sulf pre-incubation was not able to counteract the increased expression of the apoptosis regulator *BAX* gene. However it slightly increased the levels of the anti-apoptotic *BCL-2* (Supplementary Fig. S5).

### Bioavailable (poly)phenol metabolites reduce neuro-inflammation

**Figure 4:**
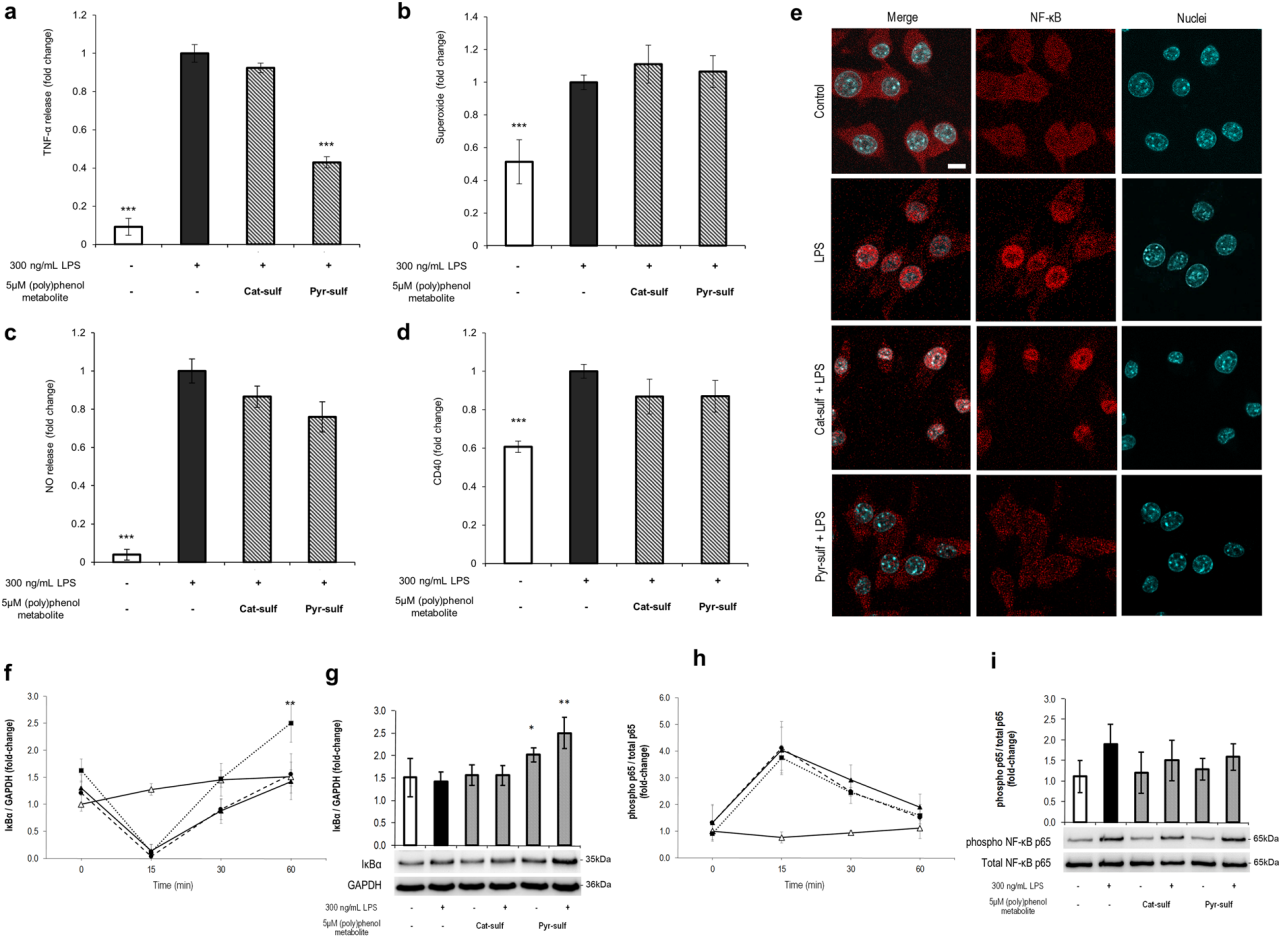
Effects on neuroinflammation by Cat-sulf and Pyr-sulf. Pro-inflammatory markers were evaluated, namely (**a**) TNF-α release, (**b**) intracellular superoxide production, (**c**) nitric oxide, and (**d**) CD40 quantified in N9 microglial cells. Cells were pre-incubated for 6 h with each of the bioavailable (poly)phenol metabolite and then challenged with 300ng/mL of LPS. Statistical differences are denoted as ***p < 0.001, **p < 0.01 and *p < 0.05 relatively to lesion (LPS). (**e**) Microglial NF-κB p65 translocation into the nucleus after 60 minutes of LPS stimulation. Cells were pre-treated with Cat-sulf or Pyr-sulf for 6 h before LPS-stimulation. NF-κB (red); Nuclei (blue) stained with DAPI. Each capture is representative of at least 3 independent biological replicates. Scale bar: 10 µm. (**f–i**) Microglial NF-κB p65 phosphorylation ratio and IκBα fold change in protein levels. (**f**) IkBα protein levels along time after LPS stimulation and (**g**) after 60 min of LPS stimulation with representative western blots. (**h**) NF-κB activation profile along time after LPS stimulation looking at NF-κB p65 phosphorylation (ser536) ratio along time after LPS stimulation and (**i**)after 60 min of LPS stimulation ﻿with representative western blots.﻿ Cells were pre-treated either with Pyr-sulf or Cat-sulf before LPS stimulation. Control cells (white triangles, solid line), LPS-stimulated cells (black triangles, solid line), cells treated with Cat-sulf prior to LPS stimulation (black circles, dashed line), cells treated with Pyr-sulf prior to LPS stimulation (black squares, dotted line). Statistical differences are denoted as *p < 0.05 and **p < 0.01 relatively to lesion (LPS). Western blots were analyzed under the same experimental conditions. Data are presented as the means ± SD, n = 3.

Neurodegenerative processes are also closely associated with neuro-inflammatory responses which are mainly mediated by the resident brain immune cells, microglia. Microglia pro-inflammatory activation was achieved by stimulation with lipopolysaccharide (LPS)^42^ and the effect of pre-incubation with metabolites prior to LPS insult was studied (Fig. 4 and Supplementary Fig. S﻿﻿﻿﻿﻿﻿6). We measured several parameters related to inflammatory responses such as reactive oxygen and nitrogen species (ROS/RNS), TNF-α inflammatory cytokine and CD40 expression. We found that pre-incubation with Pyr-sulf significantly inhibited TNF-α release (~2 fold) upon LPS stimulation (Fig. 4a) but no other markers of activation were affected by Pyr-sulf (Fig. 4b,c and d). These results suggest that Pyr-sulf specifically affects TNF production. Nevertheless, some of the other less abundant bioavailable (poly)phenol metabolites also gave positive effects for the four pro-inflammatory markers, suggesting that each compound may have different potential (Supplementary Fig. S6).

### Pyr-sulf prevents NF-κB nuclear translocation and alters IκBα levels

TNF-α levels are mainly regulated by the NF-κB pathway^43^. To investigate the mechanisms underlying the inhibitory effect of Pyr-sulf on TNF production, we analyzed NF-κB activation. We followed NF-κB translocation to the nucleus and its phosphorylation levels on ser536. In addition, we analyzed the kinetics of IκBα degradation, which inhibits NF-κB translocation to the nucleus^43^.

After one hour, LPS induced a clear nuclear translocation of NF-κB to the nucleus of N9 cells (Fig. 4e). However, with Pyr-sulf but not Cat-sulf pre-incubation﻿, LPS-stimulated NF-κB translocation to the nucleus in microglia is not observed at this time point﻿ (Fig. 4e). Similar results were obtained with primary cultures of rat microglia (Supplementary Fig. S7). N9 cells treated with LPS showed, as expected, a significant decrease in IκBα levels after 15 min when compared to non-treated cells (Fig. 4f). Pre-incubation with Pyr-sulf did not prevent IκBα reduction at 15 min, but it boosted the recovery of IκBα levels after 30–60 min of LPS treatment (Fig. 4f and g). Interestingly, Pyr-sulf alone enhanced IκBα basal levels, even without an inflammatory stimulus (Fig. 4g and Supplementary Fig. S8). The NF-κB phosphorylated-p65/total p65 ratio peaked at 15 min (Fig. 4h **)** and then decreased to basal cellular levels at 60 min (Fig. 4h and i). This kinetics was unchanged by pre-treating either with Pyr-sulf or Cat-sulf (Supplementary Fig. S8). Overall, our data suggest that Pyr-sulf pre-treatment increased the stability of IκBα rather than inhibiting NF-κB phosphorylation.

## Discussion

Dietary (poly)phenols present neuroprotective potential but their selective permeability across BBB, poor absorption, rapid metabolism and systemic elimination limit their bioavailability and could limit their protective efficacy^21, 27^.

Taking advantage of a robust, though simplified, *in vitro* model of the human BBB which has been validated for CNS drugs^32, 44, 45^, we confirmed that plasma-bioavailable (poly)phenol metabolites could be transported across the BBB endothelium. The differences in endothelial transport for the metabolites could be related with their degree of chemical modification: methylation combined with sulfation enhanced the transport of gallic acid and catechol derivatives, but the same was not true for pyrogallol derivatives. In fact, Youdim and colleagues noted that transmembrane diffusion of some (poly)phenol metabolites *in vitro* was related to their lipophilicity, where less polar (e.g. methylated) derivatives achieved greater uptake than more polar derivatives (e.g. sulfates and glucuronides)^28, 29^. Nevertheless, it is not clear whether the primary route by which (poly)phenols metabolites cross the BBB is by simple diffusion or by carrier-mediated transport.

Our data for BBB endothelial transport can be partially explained by the *in silico* predictions. For instance, 4-MeCat-sulf, the metabolite with the higher endothelial transport percentage, also presented the higher QPlogPo/w. Moreover, for this metabolite and for Cat-sulf, the lowest PSA values were reported, below the recommended threshold of 90Å^2^ to be considered suitable for passive BBB permeation^33^. It is important to highlight that the descriptors calculated by the *in silico* analysis have to be regarded as whole and are integrated only assuming passive BBB diffusion. However, the contribution of active transport and metabolic transformation should not be discounted. In fact, the chemical ability of some metabolites to establish hydrogen bonds (indicated by some descriptors) with other functional groups of biomolecules (*e.g.* amine and hydroxyl groups of proteins like albumin) could suggest a propensity for an active transport mechanism in BBB. Nevertheless, such descriptors do not necessarily imply that the studied molecules are inactive in the brain, as there are other ways to transport molecules across the BBB, as it is seen for very polar essential molecules such as hexoses, amino acids and several orally administered medicines and other drugs. Overall our experimental results, together with the *in silico* predictions, suggest that transport across BBB could not be simply justified by passive permeation and some form of active transport must also be involved.

Interestingly, HBMEC cells seemed to favor the transport of one isomer of Pyr-sulf, Pyr-2-Sulf, which was also the main form found in human plasma^22, 30^. Even after an extended incubation period (24 h), no transport of the other isomer, Pyr-1-sulf was detected, suggesting selective transport. This may be due to the enhanced hydrogen bonding ability of the vicinal diol structure in Pyr-2-sulf compared to Pyr-1-sulf which could influence protein binding^46^. Existence of an isomer-selective transport in endothelial cells of BBB has already been reported for (+)-catechin and (−)-epicatechin^47^, although this is a stereoisomer rather than a positional isomer. Our results suggest that HBMEC may favor the uptake of the most abundant bioavailable metabolite of a mixture in equal proportions.

The presence of efflux transporters was confirmed for the first time in HBMEC cells and these ABC-type transporters may contribute to overall BBB transport of the (poly)phenol metabolites. There was a slight but significant increase in Cat-sulf transport in the presence of a BCRP inhibitor, which suggests that this efflux transporter can limit Cat-sulf transport. In fact, other studies have also highlighted the involvement of BCRP in limiting (poly)phenol access to the brain. For instance, quercetin, when co-administrated with a P-gp or BCRP inhibitor, entered BBB epithelia but was then specifically exported by the BCRP^28^. Such phenomenon may partly explain why quercetin presents very limited bioavailability, despite its plasma bioavailability.

As well as acting as substrates for these efflux pumps, (poly)phenol metabolites can modulate the activity of ABC transporters, putatively influencing brain bioavailability of other compounds (reviewed in^48^). However, our results for P-gp activity suggest that none of the metabolites activated or inhibited this efflux pump. Therefore we propose that differential transport observed across the BBB endothelium cannot be a consequence of a differential efflux of the (poly)phenol metabolites. It should be noted that the BBB is a complex interface composed not only by endothelial cells but also by astrocytes and perycites^23, 31^, and these cell types may also influence BBB transport of (poly)phenol metabolites *in vivo*.

Taking advantage of the power of the MS analysis, we observed that HBMEC metabolized the phenolic sulfates into novel metabolites, mainly glutathione and glucuronic acid derivatives. Indeed, capillary endothelial cells are known to possess the enzymes required for these conversions, such as glutathione S-transferase, UDP-glucuronosyl-transferase^49^, gamma glutamyl transpeptidase and catechol-*O*-methyltransferase^49^. Enzymes known primarily for hepatic drug metabolism have also been shown to exist in the brain, albeit at relatively low specific activities^50^ in particular at the blood-brain interfaces where they influence cerebral availability of toxic compounds^49, 51^. An *in vitro* study to assess (+)-catechin and (−)-epicatechin permeability across the BBB detected new glucuronide derivatives using the human brain capillary endothelial cell line hCMEC/D3^47^. Similarly, Liang and co-workers^52^ performed a comprehensive LC-MS study on (+)-catechin metabolism product distribution in rat tissues and reported 3-*O*-Me-catechin-5-*O*-glucuronide was present in the brain tissue. Our studies also provided evidence for the interconversion of some metabolites into new metabolites. For example, acetylcysteine-2-*O*-methylcatechol, was detected in both Cat-sulf and Pyr-sulf samples, although in different relative amounts. Also, novel metabolites were detected in samples from gallic acid derivatives and from VA-sulf. The extensive metabolism of these simple phenolic sulfates by HBMEC reinforces the importance of studying the metabolites of (poly)phenols and not their parent compounds. We speculate that further interconversions may occur to, for example, valeric acid and hydroxyphenyl-propanol derivatives^52^ and the physiological relevance of these new metabolites is unknown. Moreover, other cell types resident in the BBB besides endothelial cells, may also contribute to the generation of different new (poly)phenol variants. Further studies with more complex BBB *in vitro* models (*e.g*. co-cultures of brain endothelial cells with astrocytes) will better elucidate potentially novel brain-targeted (poly)phenol metabolites. On the other hand, by contributing also for the synaptic function, astrocytes role in (poly)phenol metabolites supply to neurons in a neuronal *in vivo* context must be further contemplated.

The (poly)phenol metabolites caused a cytoprotective effect in the BBB cells challenged with hydrogen peroxide. Pyr-sulf, the most abundant metabolite detected in circulation^22^, was the most effective to prevent oxidative damage caused in HBMEC cells by hydrogen peroxide. BBB endothelial protection by resveratrol has been described^37^ but, to our knowledge, this is the first time that protection has been demonstrated using physiological concentrations of bioavailable (poly)phenol metabolites.

Neuroprotective effects of dietary (poly)phenols in primary neuronal cultures and in a 3D model containing neurons and astrocytes was also observed. In glutamate excitotoxicity conditions, pathologically relevant for neurodegenerative disorders, neurons are damaged by the excessive stimulation of nerve receptors through this neurotransmitter accumulation in the synaptic cleft^53^. Pyr-sulf caused significant neuroprotection in primary mouse cerebellar granule cells exposed to toxic glutamate concentrations. A similar protection was recently noted for pterostilbene, a resveratrol analog, described to be brain bioavailable, which attenuated glutamate-induced oxidative stress injury in murine hippocampal neuronal HT22 cells at physiological circulating levels^54^.

The 3D aggregates are a very robust system, with different functional cell types (neurons and astrocytes), interlinked and communicating, in a three-dimensional architecture, which is more directly related with *in vivo* environment of brain cells^41, 55^. The neuroprotection by human bioavailable (poly)phenol metabolites in these neuron-astrocyte models reinforces the pharmacological importance of these metabolites. Exposure to *t*-BHP induced greater damage to neurons than astrocytes within the 3D aggregates but Pyr-sulf did not recover the levels of the neuronal marker, βIII-tubulin and also did not affect GFAP protein levels (the astrocyte marker). Neurons have been described to have a greater susceptibility to oxidative injury as compared to astrocytes^56^. In fact, our 3D aggregates are mainly composed of astrocytes (~77% of the cells)^41^. Glial cells (astrocytes and microglia) are responsible for the repair process of the brain after injury^57^, being stronger and less susceptible to damage than neurons. Protection by Pyr-sulf in this system could then be related with the improving functionality of astrocytic population inside aggregates and not directly reducing the deleterious effects of the oxidative injury. However, there is still a lack of studies demonstrating the effect of phenolic metabolites on these cells. It have already been described that resveratrol can ameliorate glutamatergic metabolism and transmission and thus synaptic plasticity and neuroprotection^58–60^. The missing link between (poly)phenol metabolites effects and neuroglial communication/signaling is therefore of upmost importance to be addressed for an effective translation of how diet can alter age-related neurological diseases, like Alzheimer’s disease.

Inflammation and neurodegeneration are normally associated in neurological conditions. Here, we found that, Pyr-sulf, besides its neuroprotective effect, also attenuated TNF-α release in microglia challenged with LPS. TNF-α, a classic and reliable marker of microglia activation, is known to be regulated by NF-κB. Dietary phenolics, such as quercetin, curcumin and resveratrol, have been shown to inhibit signaling pathways involved in microglia cells activation^61^, namely NF-κB. However, the effect of their metabolites has not yet been assessed probably due to their poor bioavailability. To our knowledge, this is the first study where dietary (poly)phenol metabolites have been shown to modulate NF-κB-mediated microglia activation. Our results show that Pyr-sulf, at physiological concentrations after 1 h of LPS, is able to modulate IκBα protein levels, either by promoting its synthesis or by decreasing its degradation, which retains NF-κB in the cytoplasm and prevents NF-κB-dependent gene transcription. Indeed, this could explain the reduction in TNF-α levels in LPS-stimulated microglia pre-incubated with Pyr-sulf.

Taken together, these data provide new insights for the (poly)phenol metabolites to be further explored in biochemical pathways and validated *in vivo* using appropriate animal models. Although (poly)phenol metabolites could impact on brain health and cognition indirectly through peripheral and cerebrovascular blood flow improvement, this work highlights their potential to have direct effects towards neuronal cells. The phenolic sulfate metabolites studied are transported across endothelial cells of the BBB and metabolized to form new chemical entities with their own potential biological effects. While we cannot predict the biological role/relevance of these biotransformations, the fact that BBB cells modify (poly)phenols metabolites is of vital importance for further studies. These biotransformation processes by HBMEC may facilitate metabolite elimination from brain or, on the other hand, such modifications may enhance retention or assist further uptake and delivery to other neuronal cell types, therefore spreading their beneficial effects. Moreover, since the BBB is the first line of defense of the brain and is a crucial preventive factor of neurological diseases, ensuring BBB endothelium protection through diet could have wide-spread consequences in the body. The neuroprotective potential of these phenolic sulfates, used at physiologically relevant concentrations, comparable to those detected in human plasma^22^, reflects their importance as modulators of cell metabolism and the ultimate importance of dietary habits to health and disease progression. Moreover, our studies on the abundant serum-bioavailable metabolite, Pyr-sulf, emphasized the potential pleiotropic neuroprotective effects of these phenolic metabolites. The marked reduction in TNF-α release may be associated with the neuroprotective power of Pyr-sulf: the capacity of Pyr-sulf to pre-condition cells, interfering with NF-κB nuclear translocation and being able to better respond to oxidative stimuli, an excitotoxicity burst or an inflammatory situation, confirms the potential of these human bioavailable (poly)phenol metabolites to mitigate in the intricate complexity of a neurodegenerative disorder.

## Methods

### Reagents

All the used chemicals were purchased from Sigma-Aldrich, unless stated otherwise. Acetonitrile (ACN, LC-MS grade) was purchased from Fisher Scientific Ltd. (Leicestershire, UK). LC-MS grade water was produced by an Elix/MilliQ purification system (Millipore, Waterford, UK). 4-Methylgallic acid and 2-methylpyrogallol were obtained from Apin chemicals and taxifolin was obtained from Extrasynthese. The synthesized compounds were: 4-methylgallic acid-3-*O*-sulfate (4-MeGA-sulf, 57% yield, 79% purity, containing 6% of 4-methylgallic acid and 13% of 4-methylgallic acid-3,5-*O*-disulfate), 4-methylcatechol-*O*-sulfate (4-MeCat-sulf, 66% yield, mixture of two compounds indistinguishable, pure), vanillic acid-4-*O*-sulfate (VA-sulf, quantitative yield, pure), catechol-*O*-sulfate (Cat-sulf, 66% yield, pure), pyrogallol-*O*-sulfate (Pyr-sulf, 75% yield, mixture of two compounds in equal proportions), 1-methylpyrogallol-*O*-sulfate (1-MePyr-sulf, mixture of two compounds in equal proportions, 58% yield) and 2-methylpyrogallol-*O*-sulfate (2-MePyr-sulf, 44% yield, 89% purity, containing 11% of 2-methylpyrogallol-*O*-disulfate). Synthesized compounds were obtained as sodium salts and were firstly dissolved in DMSO (Fluka) before dilution to final concentration in specific cell media (see Table 1).

### Cell culture conditions

#### HBMEC line

Human brain microvascular endothelial cell (HBMEC) line was used as a simplified and validated *in vitro* model of the BBB^32, 44, 45, 62^. This cell line was derived from primary cultures of HBMEC transfected with SV40 large T antigen^63^. HBMEC line was cultured in RPMI 1640 medium (Sigma-Aldrich) supplemented with 10% fetal bovine serum (FBS - Biochrom AG), 10% NuSerum IV (BD Biosciences), 1% non-essential amino acids (NEAA - Biochrom AG), 1% minimal essential medium (MEM) vitamins (Biochrom AG), 1 mM sodium pyruvate (Biochrom AG), 2mM L-glutamine (Biochrom AG), and 1% antibiotic-antimycotic solution (Sigma-Aldrich). For immunostaining and cytoprotection studies, cells were seeded at a density of 8 × 10^4^ cell/mL in 24-well and 96-well plates, respectively, and treated after 48 h in culture. For integrity and transport studies, cells were seeded on polyester transwell inserts (0.4 μm, Corning Costar Corp., USA) at a density of 8 × 10^4^ cell/insert and treated after 8 days in culture. Inserts and plates were coated with rat-tail collagen-I (BD Biosciences, Erembodegem, Belgium) before seeding. All experiments were performed after monolayer formation.

#### Mouse cerebellar granular cells

Primary cultures of cerebellar granule cells were prepared according to the already described method^38^. Cells were isolated from cerebella of 7d-old BALB-C mice and 0.5 × 10^6^ cell/mL were cultured in Neurobasal Medium (Gibco) supplemented with 2% B-27 without antioxidants (Gibco) and 2% KCl (20 mM, Sigma-Aldrich), containing 0.25% L-glutamine (200 mM, Sigma-Aldrich) and 0.48% penicillin-streptomycin (P/S, 5000 U mL^−1^, Gibco). Experiments were performed on 24-well plates with coverslips coated with 50 µg/mL poly-D-lysine (Sigma-Aldrich). 20 µM of cytosine arabinoside (Sigma-Aldrich) was added 48 h after inoculation to prevent glia cell proliferation. All the experiments were performed between days 7–11 in culture.

#### N9 murine microglial cell line

The N9 murine microglial cell line was kindly provided by Dr. Teresa Faria Pais. Cells were cultured in EMEM (Eagle Minimum Essential Media, Sigma-Aldrich) supplemented with 10% FBS (Gibco), 200 mM L-glutamine (Sigma-Aldrich), 1% NEAA (Sigma-Aldrich) and maintained at 37 °C, 5% CO_2_. Cells were detached by agitation before suspension of the culture media with a pipette (no cellular detaching agent was used).

#### NT2 cell line

NTera-2/cl.D1 (NT2) cells, obtained from American Type Cell Culture Collection (ATCC), were differentiated in stirred suspension culture systems, as 3D aggregates, as previously described^41^. A single cell suspension of undifferentiated NT2 cells was seeded in DMEM (Dulbeco Minimum Essential Media), 10% FBS, 1% P/S (all from Invitrogen), in a 125 mL spinner vessel equipped with ball impeller (Wheaton). After three days of aggregation, differentiation was induced by addition of 10 µM retinoic acid (Sigma-Aldrich), for three weeks, with a 50% medium exchange performed every 2–3 days. Following this period (from day 24 onwards), the medium was composed by DMEM, 5% FBS, 1% P/S. Stable 3D co-cultures of neurons and astrocytes were maintained up to day 50 of culture and applied in neuroprotection assays (from day 38 to day 50).

### Immunofluorescence

Both HBMEC and N9 cells were grown in 24-well plates with coated coverslips and immunostaining performed as already described^62, 64^. Briefly, HBMEC coverslips were incubated overnight at 4 °C with primary antibodies anti-P-gp (1:50, Calbiochem), anti-MRP1 (1:100, Millipore) and anti-BCRP (1:100, Millipore) and N9 cells coverslips were incubated overnight at 4 °C with rabbit polyclonal anti-NF-κB p65 (C-20) (1:200, Santa Cruz Biotechnology). Incubation with secondary antibodies Alexa 594 anti-rabbit IgG (1:500) and Alexa 488 anti-mouse IgG (1:500) (Invitrogen) lasted for 2 h at room temperature. Nuclei were counterstained with DAPI. Between incubations cells were washed three times with PBS. HBMEC staining was examined using a Leica DFC 490 camera (Leica, Germany) adapted to an AxioScope.A1 microscope (Zeiss, Germany), ZEN 2012 blue edition software by Carl Zeiss Microscopy GmbH, 2011. Confocal fluorescent Z-series N9 cells were acquired using on a Leica SP5 live upright confocal (Leica, Wetzlar), using a 63 × 1.3NA oil immersion objective, the UV lamp and DPSS 561 nm yellow-green laser. Post-acquiring treatment was performed using ImageJ software (NIH, USA).

### Efflux transporters functional assays

Activity of each efflux transporter was determined by measuring cellular accumulation of respective substrate^65, 66^, and results were expressed as fold-change compared to the respective control. Activity of P-gp, MRP1 and BCRP were determined by measuring the cellular accumulation of the substrates Rhodamine 123 (Sigma-Aldrich), Calcein-AM (Santa Cruz Biotechnology), and BODIPY-FL Prazosin (Life Technologies), respectively. HBMEC monolayers were washed and incubated for 1 h at 37 °C with Ringer–Hepes solution (118 mM NaCl, 4.8 mM KCl, 2.5 mM CaCl_2_, 1.2 mM MgSO_4_, 5.5 mM D-glucose, 20 mM Hepes, pH 7.4) containing 10 mM of each substrate, separately. The solution was quickly removed, HBMEC were washed three times with PBS and solubilized in 0.1 M NaOH. Substrate content was determined using a FLUOstar Omega fluorescent plate reader (BMG Labtechnologies, Ortenberg, Germany; for P-gp, excitation at 505 nm, emission at 534 nm; for MRP1, excitation at 495 nm, emission at 516 nm; for BCRP excitation at 503 nm, emission at 512 nm). A reference efflux transporter inhibitor was used as positive control: P-gp inhibitor, Verapamil (1 µM, Sigma-Aldrich); MRP1 inhibitor, MK-571 (1 µM, Santa Cruz Biotechnology); and BCRP inhibitor, Ko 143 (1 µM, Santa Cruz Biotechnology). Protein content was evaluated by the Bradford method^67^ using Bio-Rad’s Protein Assay reagent (Bio-Rad).

### BBB integrity

#### Trans-endothelial electrical resistance (TEER)

TEER was evaluated as reported previously^62^. Briefly, TEER readings were performed using an End Ohm™ chamber coupled to an EVOMX resistance meter (World Precision Instruments, Inc., USA). Readings were collected before the addition of bioavailable (poly)phenol metabolites and at the end of the incubation time. TEER was calculated as percentage of variation from average control readings, after deducting the values of empty insert.

#### Paracellular permeability

To evaluate selective paracellular permeability of the HBMEC monolayer after exposure to bioavailable (poly)phenol metabolites, a permeability assay was conducted with sodium fluorescein (molecular weight: 376 Da). The permeability was determined as described before^62^. The endothelial permeability coefficient *Pe* was calculated as described^68^ as a percentage of variation from control. In all the assays, monolayer integrity was monitored by TEER and sodium-fluorescein paracellular permeability, which confirmed that the passage of metabolites across BBB cells was not due to a disruption of the barrier properties.

### Transport Assays

HBMEC were plated in semi-permeable membranes (inserts) placed in cell culture wells. This two-chamber system, where the upper and lower chambers mimic the blood and brain compartments, respectively, and the confluent HBMEC monolayer represents the BBB. Transport assays were conducted in HBSS (Hank’s Balanced Salt Solution) with calcium and magnesium (Gibco), supplemented with 0.1% FBS. Confluent monolayers of HBMEC were incubated with 5 μM of each compound for 2 h, time described for compounds to interact and/or being transported across cells^26, 69^. In order to evaluate a time-dependent transport, 5 μM of Cat-sulf and Pyr-sulf were added to the upper site and samples from upper and lower site were collected after 2, 6 and 24 h. To evaluate efflux transporters influence in the BBB transport of the bioavailable (poly)phenol metabolites, co-incubation of 5 μM of Cat-sulf and Pyr-sulf with inhibitors of P-gp, (1 μM Verapamil), of MRP1 (1 μM MK-147) and of BCRP (1 μM Ko 143) was also performed. Monolayer integrity was ensured in all experiments as described. In the end, cell medium from upper and lower compartments were collected and frozen at −80 °C until analysis. Transport analysis were performed after sample deproteinization by LC-Orbitrap MS as described below.

#### Sample deproteinization

To 1 mL of upper or lower compartment cell medium it was added: 139 µL of 50% formic acid, ascorbic acid (final concentration of 1 mM) and taxifolin as internal standard (final concentration of 9 µM). To precipitate proteins, 2.5 mL of ACN was added dropwise and samples were vortexed before centrifugation at 3200 *g* for 15 min. The supernatant was removed and dried under centrifugal evaporation (CentriVap Vaccum Concentrator, Labconco). Samples were dissolved in 5% ACN in 0.1% formic acid and immediately analyzed by LC-Orbitrap MS.

#### LC-Orbitrap MS

Samples were separated on an HPLC Accela 600 HPLC system (Thermo Scientific, Bremen, Germany) using a C18 Synergi Hydro RP18 column (Phenomenex, Macclesfield, UK) 4 μm particle size and dimensions 2 mm ID × 150 mm. Column was fitted with a Security GuardTM guard system containing an Aqua 10 μm C18 Guard Cartridge (2 mm ID × 4 mm; Phenomenex) and eluted over a gradient of 98% solvent A (0.1% formic acid in ultra-pure water) to reach 5% B (0.1% formic acid in ACN) at 5 min, 35% B at 25 min, increase to 100% B at 26 min, 100% B at 29 min, and back to 2% B at 30 min at a flow rate of 0.2 mL/min. Analysis was done on an LTQ OrbitrapTM XL hybrid mass spectrometer (Thermo Scientific, Bremen, Germany).

MS analysis was performed using data-dependent *N*th order double play analysis comprising full scan mass range 80–2000 amu, 30 000 resolution, data-type centroid and data dependent MS/MS (60 s of exclusion duration) on the top three most intense ions detected above threshold automatically in the independent scan event. ESI settings were as follows: source voltage, 3.4 kV; capillary temperature was 275 °C with a sheath gas at 40 psi and auxiliary gas at 5 psi. MS data handling software (Xcalibur QualBrowser software, Thermo Electron Corp.) was used to search for predicted metabolites by their appropriate *m/z* value. All peaks were checked for *m/z* value and fragmentation products. Calibration curves, ranging from 0.3125 to 10 µM, were constructed from all the metabolites, and each concentration point was injected in triplicate. Standard curves were all linear within the concentration range and linearity was ensured as R^2^ 0·997–1·000. Limit of quantification was determined by analysis in triplicate of standards at low concentrations, and was defined as signal: noise ratios of 1:10. Endothelial transport was calculated as percentage determined by the ratio of lower compartment concentration and the sum of upper and lower compartments concentrations.

Search for novel compounds resulting from HBMEC metabolization was performed in the same samples using Xcalibur QualBrowser software using an in-house database of putative bioavailable (poly)phenol metabolites^30^. Putative ID of predicted metabolites was determined by exact mass, according to their appropriate *m/z* value.

### *In silico* prediction of pharmacokinetic properties

Maestro software (Schrödinger, Release 2015–4, LLC, New York, 2014) was used to create a three-dimensional computer models of studied compounds. The global minimum geometry was used as an input for the QikProp application (Schrödinger Release 2015–4, LLC, New York, 2014) to estimate various theoretical descriptors relevant for compound permeability through the BBB^70^. From QikProp pharmaceutically-relevant properties, the following parameters were retained for evaluation: octanol/water partition coefficient; predicted brain/blood partition coefficient; apparent Caco-2 or MDCK cell permeability; predicted CNS activity; Van der Waals surface area of polar nitrogen and oxygen atoms and carbonyl carbon atoms (PSA); prediction of binding to human serum albumin.

### Protective potential of human bioavailable (poly)phenol metabolites

#### Cytoprotection in HBMEC

HBMEC were incubated in the presence of H_2_O_2_ (Sigma-Aldrich). Briefly, 24 h after seeding, cells growth medium was removed and cells were pre-incubated for 24 h with 5 µM of each compound. After pre-incubation, media was replaced by new media containing 300 µM H_2_O_2_, for 24 h^71, 72^. In the end, medium was removed and cell viability was assessed using the CellTiter-Blue^®^ Cell Viability Assay (Promega), according to the manufacturer’s instructions.

#### Neuroprotection in cerebellar granule cells

Physiological concentrations (5 µM) of the two most abundant metabolites were added to the cells at 7 d in culture. Cells were then incubated for 24 h and morphology was evaluated. At day 8 in culture, 100 µM of glutamate (Sigma-Aldrich) was added to the respective wells. Cells were incubated for another 24 h, morphology was evaluated and viability determined by fluorescence microscopy through Propidium Iodide (PI) and Hoechst 33342 staining, as previously described^38^.

#### Neuroprotection in 3D co-cultures of neurons and astrocytes

Aggregates were collected between days 38 and 50, distributed in 12- or 96-well plates and cultured in DMEM, 5% FBS, 1% P/S. Human bioavailable (poly)phenol metabolites (5 µM) were added to the cultures and 24 h after *tert*-buthyl hydroperoxide (*t*-BHP; Sigma-Aldrich) oxidative lesion was induced, for a cell viability reduction of approximately 50%, in 48 h^41^. Cell viability was accessed before exposure to test compounds, before *t*-BHP lesion induction and 48 h after lesion induction by PrestoBlue® Cell Viability Reagent (Thermo Fisher Scientific), accordingly to manufacturer’s instructions. Idebenone (provided by Grupo Tecnimede) was used as positive control. Final cell viability was calculated as a percentage of cell viability before lesion induction.

#### Attenuation of neuroinflammation

N9 microglial cells were plated into 6-well plates (5 × 10^5^ cells mL^−1^) and then pre-incubated with 5 µM of each (poly)phenol metabolite. After 6 h, medium was discarded and cells were washed once with phosphate buffer saline (PBS) prior to addition of fresh medium with 300 ng/mL lipopolysaccharide from *Escherichia coli* 055:B5 (LPS, Sigma–Aldrich). Pro-inflammatory mediators release to medium were quantified as described below.

#### NO release

The NO release to media was quantified according to^73^ by Griess Reagent (Sigma–Aldrich) according to manufacturer’s instructions.

#### TNF-α quantification by ELISA

Cell supernatants were harvested after 24 h and stored at −80 °C until analysis. Murine TNF-α release was assayed by sandwich ELISA according to the manufacturer’s instructions (PeproTech^®^; Princeton Business Park, Rocky Hill NJ, United States)^74^. All the reagents and plates used were provided in the kit. The plate was incubated at room temperature in a Synergy HT microplate reader (Biotek^®^, Winooski, USA) for 35 min, with 5-min intervals Abs_405_ readings.

#### CD40 and superoxide (O_2_^•−^) quantification by flow cytometry

Culture media was discarded and PBS was added to detach N9 adherent cells, which were then incubated with mouse anti-FcγR (same as CD-16/32, from E-Biosciences) in FACS buffer (PBS containing 2% FBS and 0.01% NaN_3_) for 30 minutes at 4 °C before staining. Cells were spun down at 1000 *g*, washed once with FACS buffer and stained with 5 µg/mL mouse anti-CD40 - FITC (clone 3/23, from BD Biosciences^®^); and with 5 µg/mL DHE probe (Dihydroethidium, Invitrogen™, Carlsbad, CA, USA) as superoxide indicator^75^. Events were acquired using CUBE 6 cytometer, from Partec^®^. Post-acquisition analysis was done with the software FSC express 4 flow research edition^®^.

### Western blot

NT2 aggregates and N9 protein samples western blot analysis was performed accordingly to^41^. Aggregates were lysed with TX-100 lysis buffer (50 mM Tris, 5 mM EDTA, 150 mM NaCl, 1% Triton X-100, pH 7.4) and N9 protein extraction was performed with RIPA buffer. Primary antibodies were incubated overnight at RT, followed by secondary antibodies (horseradish peroxidase-conjugated, ECL anti-mouse IgG, anti-rat IgG or anti-rabbit IgG; Pierce, Millipore and GE Healthcare), incubated for 2 h at room temperature. Anti-GAPDH antibody (Thermo Scientific) was used as loading control. Primary antibodies used for protein detection were: anti-βIII-Tubulin (Millipore), anti-GFAP (DAKO), and MitoProfile Total OXPHOS WB primary antibody cocktail (Abcam), anti-phospho-NF-κB p65 (ser536) antibody (Cell Signalling), anti-NF-κB p65 (C-20) (Santa Cruz Biotechnology), and anti-IκB-α (C-21) antibody (Santa Cruz Biotechnology). Membranes were developed using Amersham ECL Prime Western Blotting Detection Reagent (GE Healthcare) and visualized using a ChemiDocTM XRS + System (BioRad).

### Real-time quantitative PCR

Real-time quantitative PCR analysis (qRT-PCR) was performed as described in^76^. Briefly, total RNA was extracted with High Pure RNA Isolation kit (Roche) and reverse transcription performed with Transcriptor High Fidelity cDNA Synthesis kit (Roche). qRT-PCR analysis was performed in a LightCycler 480 Multiwell Plate 96 (Roche), using the Light-Cycler 480 SYBR Green I Master Kit (Roche). cDNA was diluted 1:2 and each sample was performed in triplicates. The list of used primers and its sequence is presented in Supplementary Table S5. Cycles threshold (Ct’s) and melting curves were determined using LightCycler 480 software, version 1.5 (Roche) and results were processed using the 2^−ΔΔCt^ method for relative gene expression analysis^76, 77^. Changes in gene expression were normalized using the house-keeping gene RPL22 (coding for ribosomal protein L22) as internal control.

### Statistical analysis

The results reported in this work are the averages of at least three independent experiments and are represented as the means ± SD. Differences amongst treatments were detected by analysis of variance with the Tukey HSD (honest significant difference) multiple comparison test (a = 0.05) using SigmaStat 3.10 (Systat) software.

### Ethics statement

The research presented involves primary cultures of both cerebellar granule cells and microglial cells fundamental to the validation and understanding of phenolic metabolites in cell biology. All the procedures were performed in accordance with the guidelines and regulations under the DGAV approved license (0421/000/000/2013) by researchers accredited by Federation of European Laboratory Animal Science Associations (FELASA)/Direcção Geral de Alimentação e Veterinária (DGAV). Animals used for primary cultures extraction purpose were provided by “Instituto Gulbenkian de Ciência” Animal Facility in compliance with the Portuguese and European laws (Directive 2010/63/EU on the protection of animals used for scientific purposes), under regulation of the Portuguese official Veterinary Directorate (DGAV), which complies with the European Directive and follows the FELASA guidelines and recommendations concerning laboratory animal welfare.

## Electronic supplementary material

Supplementary Figures S1-S7 and Supplementary Tables S1-S5
