# Correction to: Polyphenols journey through blood-brain barrier towards neuronal protection

**DOI:** 10.1038/s41598-021-96179-w

**Published:** 2021-08-18

**Authors:** I. Figueira, G. Garcia, R. C. Pimpão, A. P. Terrasso, I. Costa, A. F. Almeida, L. Tavares, T. F. Pais, P. Pinto, M. R. Ventura, A. Filipe, G. J. McDougall, D. Stewart, K. S. Kim, I. Palmela, D. Brites, M. A. Brito, C. Brito, C. N. Santos

**Affiliations:** 1 Instituto de Tecnologia Química e Biológica-António Xavier, Universidade Nova de Lisboa, Av. da República, EAN, 2781-901 Oeiras, Portugal; 2 Instituto de Biologia Experimental e Tecnológica, Apartado 12, 2781-901 Oeiras, Portugal; 3 Instituto Gulbenkian de Ciência, Rua da Quinta Grande, 6, 2780-156 Oeiras, Portugal; 4 Escola Superior Agrária, Instituto Politécnico de Santarém, Qta do Galinheiro, Santarém, Portugal; 5 Medical Department, Grupo Tecnimede, 2710-089 Sintra, Portugal; 6 The James Hutton Institute, Invergowrie, Dundee, DD2 5DA Scotland, UK; 7 Engineering and Physical Sciences, Heriot Watt University, Edinburgh, EH14 4AS Scotland, UK; 8 NIBIO, Norwegian Institute of Bioeconomy Research, Pb 115, NO-1431 Ås, Norway; 9 Division of Infectious Diseases, Johns Hopkins University School of Medicine, 600 North Wolfe Street Park 256, Baltimore, MD 21287 USA; 10 Research Institute for Medicines (iMed.ULisboa), Faculty of Pharmacy, Universidade de Lisboa, Av. Prof. Gama Pinto, 1649-003 Lisbon, Portugal; 11 Department of Biochemistry and Human Biology, Faculty of Pharmacy, Universidade de Lisboa, Av. Prof. Gama Pinto, 1649-003 Lisbon, Portugal

## Introduction

Correction to: *Scientific Reports* 10.1038/s41598-017-11512-6, published online 13 September 2017

The original version of this Article contained an error in Figure 3a where the oxidative lesion applied was incorrect,

“300 µM t-BHP”

now reads:

“300 µM H_2_O_2_”

Moreover, the original version of this Article also contained an error in Figure 3b where the glutamate excitotoxicity was incorrect,

“300 µM H_2_O_2_”

now reads:

“100 µM glutamate”

The figure legend was correct at the time of publication. The original Figure 3 and accompanying legend appear below.

The original Article has been corrected.

**Figure 3:**
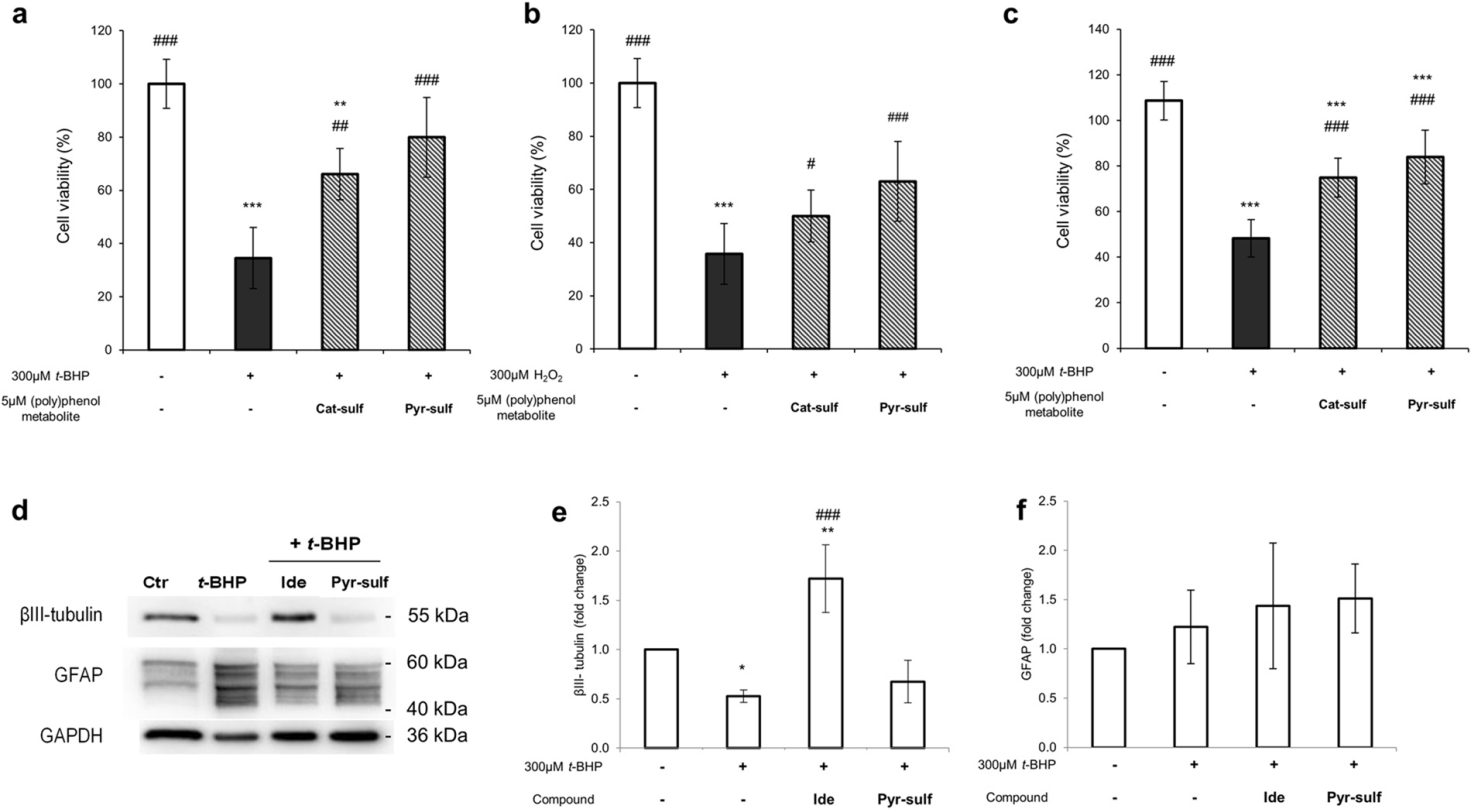
Cytoprotective potential of Cat-sulf and Pyr-sulf. (**a**) HBMEC line submitted to oxidative stress (300 µM H_2_O_2_); (**b**) primary mouse cerebellar granule cells exposed to glutamate excitotoxicity (100 µM glutamate); (**c**) 3D aggregates containing neurons and astrocytes exposed to oxidative injury (300 µM *t*-BHP). Cells were pre-incubated with 5 µM of each bioavailable polyphenol metabolite for 24 h and then injured with the respective lesion. Cell viability was assessed and is presented as percentage relatively to control. Statistical differences are denoted as ***p < 0.001, **p < 0.01 and *p < 0.05 relatively to control and as ^###^p < 0.001, ^##^p < 0.01 and ^#^ ﻿p﻿ < 0.05 relatively to each lesion (H_2_O_2_, glutamate or *t*-BHP). (**d–f**) Alterations in protein markers of the neuronal (β-III tubulin) and astrocytic (GFAP) population of 3D aggregates t﻿﻿owards the *t-*BHP lesion without and with﻿ pre-incubation with idebenone (Ide), a control drug, and with P﻿yr-sulf. (**d**) Representative western blot and (**e**) β-III tubulin and (**f**) GFAP fold changes in protein levels normalized to GAPDH. Statistical differences are denoted as ***p < 0.001, **p < 0.01 and *p < 0.05 relatively to control and as ^###^p < 0.001 relatively to *t*-BHP. Western blots were analyzed under the same experimental conditions. Data are presented as the means ± SD, n = 3.

