# Supplementary material for: Polyphenols journey through blood-brain barrier towards neuronal protection
Source: Sci Rep. 2017 Sep 13;7:11456. doi: 10.1038/s41598-017-11512-6 (PMC5597593; doi:10.1038/s41598-017-11512-6)
Supplement: Supplementary file 1 — Supplementary Figures S1-S7 and Supplementary Tables S1-S5 [file 41598_2017_11512_MOESM1_ESM.doc]

**Polyphenols journey through blood-brain barrier towards neuronal protection**

Figueira I, Garcia G, Pimpão RC, Terrasso AP, Costa I, Almeida AF, Tavares L, Pais TF,

Pinto P, Ventura MR, Filipe A, McDougall GJ, Stewart D, Kim KS,

Palmela I, Brites D, Brito MA, Brito C, Santos CN

**Supplementary Data**

**Supplementary Figure S1.** Toxicity of human bioavailable (poly)phenol metabolites in HBMEC line. Cells were pre-incubated with 5µM of each bioavailable polyphenol metabolite for 24h. Cell viability was assessed and is presented as percentage relatively to control. All values are means ± SD, n=3.

**Supplementary Figure S2.** Blood-brain barrier transport of human bioavailable (poly)phenol metabolites. Endothelial transport of 5μM (white) or 10μM (black) of each human bioavailable polyphenol metabolite after 2h of incubation. Endothelial transport was evaluated by LC-Orbitrap MS and is presented as percentage (%) determined by the ratio of the lower compartment concentration and the sum of the upper and lower compartments concentrations.

***

**Supplementary Figure S3.** P-glycoprotein substrate accumulation for (poly)phenol metabolites tested. Intracellular accumulation of P-gp substrate, Rhodamine 123 was evaluated by fluorescence after pre-incubation of cells with each of the bioavailable (poly)phenol metabolites compared with P-gp inhibitor, verapamil. Results are presented normalized for protein content. All values are means ± SD, n=3.

**a**

**b**

**Supplementary Figure S4.** Cytoprotective potential of human bioavailable (poly)phenol metabolites in different cell systems: (**a**) HBMEC line submitted to oxidative stress (300µM H_2_O_2_); (**b**) 3D aggregates exposed to oxidative injury (300µM *t*-BHP). Cells were pre-incubated with 5µM of each bioavailable polyphenol metabolite for 24h and then injured with the respective insult. Cell viability was assessed and is presented as percentage relatively to control. Statistical differences are denoted as *** p<0.001,
** p<0.01 and * p<0.05 relatively to control and as ###p<0.001, ##p<0.01 and #<0.05 relatively to each insult (H_2_O_2_ or *t*-BHP). All values are means ± SD, n=3.

**c**

**a**

*

**

**

#

**

#

*

#

**b**

- 55 kDa

- 48 kDa

- 40 kDa

- 30 kDa

- 20 kDa

- 36 kDa

**Supplementary Figure S5.** Alterations in oxidative stress, mitochondrial complexes and apoptosis in 3D aggregates. (**a**) Levels of gene expression of *SOD1*, *GPX1* and *GSR*, or (**c**) *BAX* and *BCL-2* in control 3D aggregates (white), 3D aggregates injured with *t*-BHP (black), 3D aggregates pre-incubated with 200nM Idebenone (Ide) and injured with *t*-BHP (light grey) and 3D aggregates pre-incubated with 5µM Pyr-sulf and injured with *t*-BHP (dark grey). Changes in gene expression were normalized using the housekeeping gene RPL22 (coding for ribosomal protein L22) as internal control. (**b**) Protein levels of mitochondrial subunits of the respiratory chain in 3D aggregates: representative western blot and corresponding fold changes in protein levels normalized to GAPDH. Statistical differences are denoted as **p<0.01 and *p<0.05 relatively to control and as #p<0.05 relatively to *t*-BHP. All values are mean (fold change relative to control) ± SD of three independent experiments.

**a**

**b**

**c**

**d**

**Supplementary Figure S6.** Human bioavailable polyphenol metabolites effects on neuroinflammation. Pro-inflammatory markers were evaluated, namely (**a)** nitric oxide, detected by Griess reaction, (**b)** TNF-α release, detected by quantitative ELISA, intracellular superoxide production (**c**) and CD40 (**d**) quantified by flow cytometry in N9 murine microglial cells. Cells were pre-incubated for 6h with each of the bioavailable (poly)phenol metabolites and then challenged with 300ng/mL of LPS. Statistical differences are denoted as ###p<0.001, ##p<0.01 and #<0.05 relatively to lesion (LPS). All values are means ± SD, n=3.


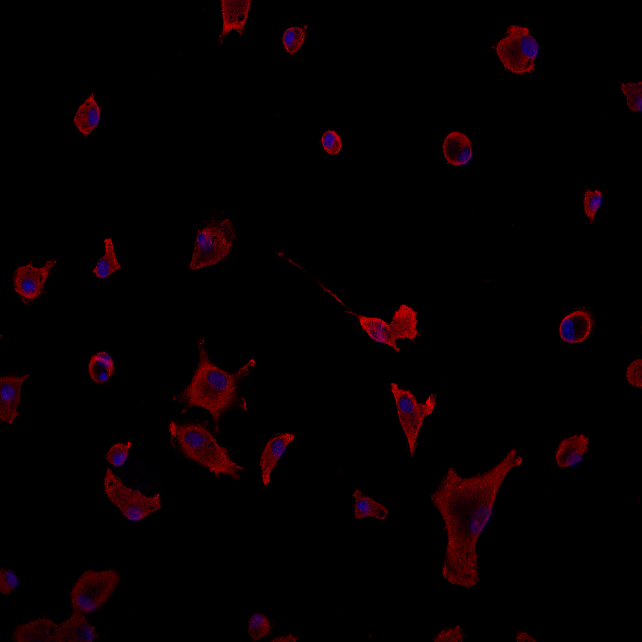


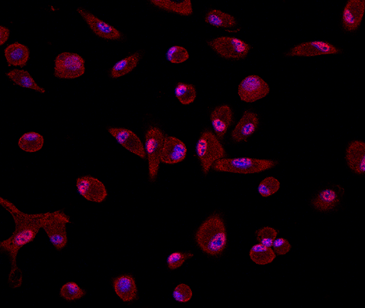

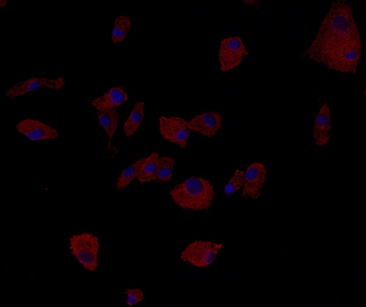


**a**

**b**

**c**

**d**


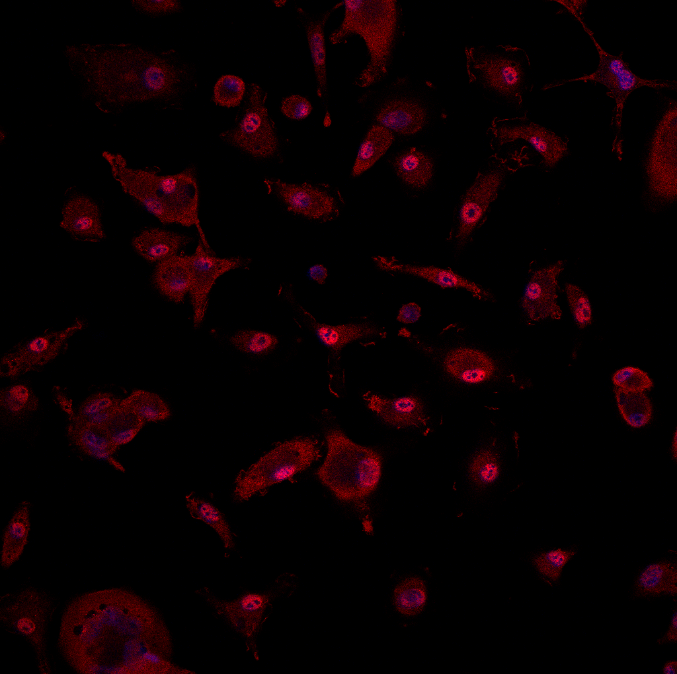


**Supplementary Figure S7.** Microglial NF-κB p65 translocation into the nucleus after 60 minutes of LPS stimulation. Immunofluorescence microscopy images of primary cultures of rat microglia*: (**a**) control cells, (**b**) microglia stimulated with LPS only, and (**c**) microglia pre-treated with Cat-sulf or (**d**) Pyr-sulf for 6 h before LPS-stimulation. NF-κB (red); Nuclei (cyan) stained with DAPI. Scale bar: 30 µm.

*** Establishment of rat primary cell cultures of microglia**

Briefly, new born Wistar rats were dissected and the heads removed. Cortices were placed into petri dishes and meninges removed. Brains were mechanically disrupted and the suspension centrifuged. Cells were cultured in high-glucose DMEM with Glutamax and supplemented with 10% FBS, 5 μg/mL insulin, 2.0 mg/mL L-glucose and 1% Pen/Strep for 14–21 days. Granulocyte-macrophage colony-stimulating factor was added at the beginning of the culture and subsequently every 3 days at a final concentration of 0.25 ng/mL. At day 14, confluent mixed glial cell cultures were shaken for 2 h at 200 rpm. Microglial cells were obtained from the supernatant after filtering through 100-μm cell strainers. Cells were re-suspended in DMEM M2279 with Glutamax supplemented with 10% FBS, 50 μM β-mercaptoethanol and 1% Pen/Strep. Cells were plated in 24 well plates for 1 h. Adherent cells were washed twice with PBS to remove cell debris and astrocytes.

Phospho NF-κB p65

Total NF-κB p65

IκBα

GAPDH

**a**

**c**

**b**

- 65 kDa

- 65 kDa

- 35 kDa

- 36 kDa

**Supplementary Figure S8.** Microglial NF-κB p65 phosphorylation ratio and IκBα protein levels for cells treated only with Cat-sulf and Pyr-sulf. (**a**) IkBα protein levels along time. (**b**) NF-κB activation profile along time looking at NF-κB p65 phosphorylation (ser536) ratio. Cells were pre-treated either with Pyr-sulf (white squares, dotted line) or Cat-sulf (white circles, dashed line). All values are means ± SD, n=3. (**c**) Representative western blot for control cells and cells treated only with (poly)phenol metabolites as indicated.

**Supplementary Table S1.** Putative new cellular metabolites arising from human bioavailable (poly)phenol metabolites in HBMEC, namely from Pyr-sulf and Cat-sulf. Using in-house database of predicted human bioavailable (poly)phenol metabolites and taking advantage of Orbitrap-MS data collected, search for novel metabolites was performed using Xcalibur QualBrowser software. Putative ID of predicted metabolites was determined by exact mass, according to their appropriate *m/z* value in both upper and lower compartment samples for each parent compound.

| **Parent compound** | **Metabolite nomenclature** | **Structure** | **Exact mass** | **m/z** | **Apical** | **Basolateral** | **Other parents compounds** |
| --- | --- | --- | --- | --- | --- | --- | --- |
| Pyr-sulf | *Glutathionyl-pyrogallol* | *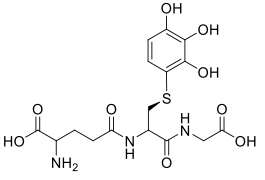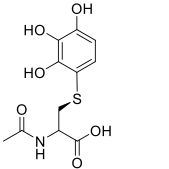* | 431,0998 | 430,4 | + | + | 1-MePyr-sulf 2-MePyr-sulf |
| Pyr-sulf | *Acetylcysteine-pyrogallol* |  | 287,0464 | 286,3 | + | + | 1-MePyr-sulf 2-MePyr-sulf |
| Pyr-sulf | *2-O-methylcatechol-O-sulfate* | *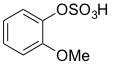* | 204,0092 | 203,2 | + | + | 1-MePyr-sulf 2-MePyr-sulf |
| Pyr-sulf | *Glutathionyl-catechol* | *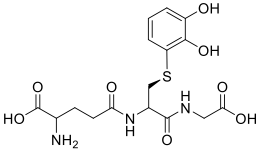* | 415,1049 | 414,4 | + | - | - |
| Pyr-sulf Cat-sulf | *Acetylcysteine-2-O-methylcatechol* | *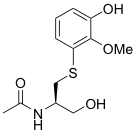* | 301,0620 | 300,1 | + | + | 4-MeCat-sulf 1-MePyr-sulf 2-MePyr-sulf |
| Cat-sulf | *2-O-methylcatechol* | *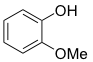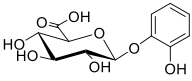* | 124,0524 | 123,1 | + | + | - |
| Cat-sulf | *Catechol-1-O-glucuronide* | *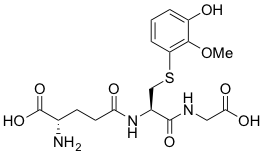* | 286,0689 | 285,2 | + | + | 4-MeCat-sulf |
| Cat-sulf | *Glutathionyl-2-O-methylcatechol* |  | 429,1206 | 428,4 | + | + | 4-MeCat-sulf |

**Supplementary Table S2.** Putative new cellular metabolites arising from human bioavailable (poly)phenol metabolites in HBMEC, namely from VA-sulf. Using in-house database of predicted human bioavailable (poly)phenol metabolites and taking advantage of Orbitrap-MS data collected, search for novel metabolites was performed using Xcalibur QualBrowser software. Putative ID of predicted metabolites was determined by exact mass, according to their appropriate m/z value in both upper and lower compartments samples for each parent compound.

| **Parent compounds** | **Metabolite nomenclature** | **Structure** | **Exact mass** | **m/z** | **Apical** | **Basolateral** |
| --- | --- | --- | --- | --- | --- | --- |
| VA-sulf | *Vanillic acid* | *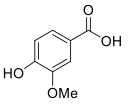* | 168,0423 | 167,2 | + | - |
| VA-sulf | *Vanillic-4-O-glucoronide* | *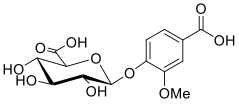* | 344,0743 | 343,3 | + | + |
| VA-sulf | *Glutathionyl-vanillic acid* | *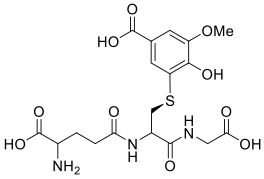* | 473,1104 | 472,4 | + | + |

**Supplementary Table S3.** Putative new cellular metabolites arising from human bioavailable (poly)phenol metabolites in HBMEC, namely from 4-MeGA and 4-MeGA-sulf. Using in-house database of predicted human bioavailable (poly)phenol metabolites and taking advantage of Orbitrap-MS data collected, search for novel metabolites was performed using Xcalibur QualBrowser software. Putative ID of predicted metabolites was determined by exact mass, according to their appropriate m/z value in both upper and lower compartments samples for each parent compound.

| **Parent compounds** | **Metabolite nomenclature** | **Structure** | **Exact mass** | **m/z** | **Apical** | **Basolateral** |
| --- | --- | --- | --- | --- | --- | --- |
| 4-MeGA  4-MeGA-sulf | *4-O-methylgallic acid-3-O-glucuronide* | *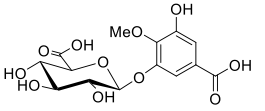* | 360,0693 | 359,3 | + | + |
| 4-MeGA  4-MeGA-sulf | *4,5-O-dimethylgallic acid-3-O-glucuronide* | *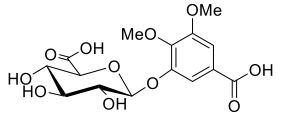* | 374,0849 | 373,3 | + | + |
| 4-MeGA  4-MeGA-sulf | *4,5-O-dimethylgallic acid-3-O-sulfate* | *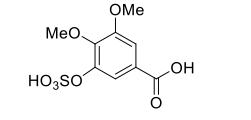* | 278,0096 | 277,2 | + | + |
| 4-MeGA-sulf | *Galloylglycine* | *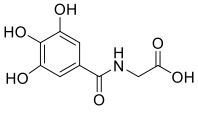* | 227,0430 | 227,2 | + | + |
| 4-MeGA  4-MeGA-sulf | *Glutathionyl-4-O-methylgallic acid* | *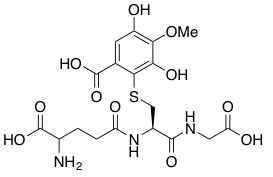* | 489,1053 | 488,4 | + | + |
| 4-MeGA | *Acetylcysteinyl-gallic acid* | *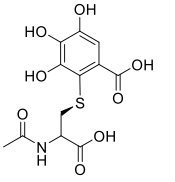* | 331,0362 | 330,3 | + | + |

**Supplementary Table S4.** Putative new cellular metabolites arising from human bioavailable (poly)phenol metabolites in HBMEC, namely from 4-MeCat-sulf, 1-MePyr-sulf and 2-MePyr-sulf. Using in-house database of predicted human bioavailable (poly)phenol metabolites and taking advantage of Orbitrap-MS data collected, search for novel metabolites was performed using Xcalibur QualBrowser software. Putative ID of predicted metabolites was determined by exact mass, according to their appropriate m/z value in both upper and lower compartments samples for each parent compound.

| **Parent compound** | **Metabolite nomenclature** | **Structure** | **Exact mass** | **m/z** | **Apical** | **Basolateral** |
| --- | --- | --- | --- | --- | --- | --- |
| 4-MeCat-sulf | *Catechol-1-O-sulfate* | *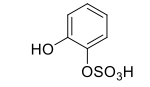* | 189,9936 | 189,2 | + | + |
| 1-MePyr-sulf 2-MePyr-sulf | *Pyrogallol-O-sulfate* | 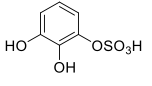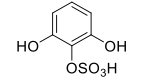 | 205,9885 | 205,2 | + | - |
| 1-MePyr-sulf 2-MePyr-sulf | *1-O-methylpyrogallol* | *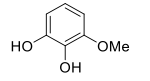* | 140,1366 | 139,1 | + | - |

**Supplementary Table S5.** List of primers used in qRT-PCR analysis.

| **Gene** | **Cell type/Function** | **Primers forward (top)**  **and reverse (bottom)** |
| --- | --- | --- |
| *RPL22* | Ribosomal protein L22 (housekeeping gene) | CACGAAGGAGGAGTGACTGG  TGTGGCACACCACTGACATT |
| *SOD1* | Superoxide dismutase 1 | AGGCCCCTTAACTCATCT  CTACAGGTACTTTAAAGCAACTCT |
| *GPX1* | Glutathione peroxidase 1 | TTTGGGCATCAGGAGAACGC  AGCATGAAGTTGGGCTCGAA |
| *GSR* | Glutathione reductase | CGTGGAGGTGCTGAAGTTCTC  TCATGGTCATGACTGGTAGCC |
| *BCL-2* | B-cell lymphoma 2 | ATCGCCCTGTGGATGACTGAG  CAGCCAGGAGAAATCAAACAGAGG |
| *BAX* | BCL-2 associated X protein | TGGAGCTGCAGAGGATGATTG  GAAGTTGCCGTCAGAAAACATG |
|  |  |  |
